# Antifungal activity of fresh ginger (*Zingiber officinale*) essential oil from ginger production waste against banana anthracnose caused by *Colletotrichum musae*: in vitro and in vivo evaluation

**DOI:** 10.1007/s42770-026-02108-2

**Published:** 2026-10-08

**Authors:** Maria Alice Brandão Silva, Aldino Neto Venâncio, Isabella da Costa Teixeira, Ana Paula Candido Gabriel Berilli, Sávio da Silva Berilli, Luciana Alves Parreira, Mario Ferreira Conceição Santos, Antônio Fernando de Souza, Luciano Menini

**Affiliations:** 1 Graduate Program in Agroecology, Federal Institute of Espírito Santo, Campus Alegre, Alegre, Espírito Santo Brazil; 2 Federal Institute of Espírito Santo, Campus Santa Tereza, Santa Tereza, Espírito Santo, Brazil; 3 Department of Chemistry and Physics, Federal University of Espírito Santo, Alegre, Espírito Santo Brazil

**Keywords:** Essential oil, Postharvest disease, Antifungal, Biocontrol, α-zingiberene, *Colletotrichum musae*

## Abstract

Banana anthracnose, caused by *Colletotrichum musae*, represents an important phytosanitary constraint, traditionally managed with synthetic fungicides, whose intensive use has contributed to environmental impacts and the selection of resistant isolates. In this context, essential oils have emerged as sustainable alternatives, especially when obtained from ginger production waste. This study aimed to characterize the chemical composition and evaluate the in vitro and in vivo antifungal activities of fresh ginger essential oil (*Zingiber officinale*) (FGEO) extracted from ginger production waste against *C. musae*. The oil was obtained by hydrodistillation and analyzed by GC-FID and GC-MS. In vitro assays were conducted at concentrations ranging from 0.039 to 0.938 µL mL⁻¹, while in vivo tests used concentrations from 0.201 to 3.212 µL mL⁻¹, including appropriate controls. Chemical analysis revealed predominance of α-zingiberene (31.73%), α-farnesene (14.85%), geranial (13.15%), and sesquiphellandrene (11.04%), with a mean extraction yield of 0.21% (w/w). The FGEO exhibited significant antifungal activity, inhibiting mycelial growth and reducing disease severity. The in vivo IC₅₀ and IC₉₀ values were 0.535 and 2.797 µL mL⁻¹, respectively, demonstrating high efficacy at low concentrations. These results support the potential of FGEO, particularly when obtained from ginger production waste, as a low-concentration, sustainable alternative for postharvest management of banana anthracnose.

**Supplementary Information:**

The online version contains supplementary material available at https://doi.org/10.1007/s42770-026-02108-2.

## Introduction

Banana (*Musa* spp.) is one of the most produced and traded fruits worldwide. The largest global producers are located in Asia, Latin America, and Africa [1]. Brazil produced 7,046,345 tonnes of bananas in 2024 [2], thereby generating employment and income for countless rural families [1]. Despite its economic and social importance, banana is susceptible to several phytopathogens, such as Colletotrichum musae, a fungus that causes anthracnose, a disease responsible for postharvest losses in fruits [3]. For its control, the application of synthetic chemical products is one of the main methods used; however, when used in excess, these products can favor the development of pathogens resistant to the mode of action and active ingredient, thereby compromising the environment and human health [4, 5].

Infection by *C. musae* generally begins when conidia germinate on the fruit surface and form appressoria that penetrate the fruit peel. The pathogen may remain quiescent in immature fruits and resume development during ripening, when anthracnose lesions become visible and rapidly compromise fruit quality. This latent infection complicates postharvest control, as symptomless fruits may already be infected at the time of harvest. Moreover, concerns regarding fungicide residues, environmental effects, and the selection of resistant fungal populations reinforce the need for safe, plant-derived alternatives for postharvest disease management [4].

Postharvest anthracnose is commonly managed using synthetic fungicides, including benzimidazole products, such as thiabendazole. Repeated reliance on fungicides with the same mode of action may select for resistant fungal populations and increase concerns regarding residues and environmental exposure [4, 5]. Therefore, plant-derived products, including essential oils (EOs), have been investigated as complementary postharvest treatments [4, 6–9]. Ginger (*Zingiber officinale*) EOs have demonstrated antioxidant [10], antibacterial [11], insecticidal [12], and antifungal activities [13, 14]. These biological properties have also increased interest in the valorization of residues generated during ginger production and postharvest processing as alternative sources of bioactive compounds. In this context, ginger production waste comprises fresh unprocessed rhizomes and rhizome fragments rejected during postharvest cleaning and grading because they do not meet commercial quality standards.

These residues have no commercial destination and are typically discarded or left in the production area as organic matter. The valorization of this material as a source of bioactive products may therefore reduce waste disposal while adding value to the ginger production chain. Despite this potential, ginger production waste remains insufficiently investigated as a feedstock for obtaining essential oils with antifungal activity [4, 14, 15]. Therefore, this study characterized the chemical composition of fresh ginger essential oil obtained from ginger production waste and evaluated its in vitro and in vivo antifungal activity against *C. musae*, the causal agent of banana anthracnose.

## Materials and methods

### Materials

Ginger used in this study consisted of ginger production waste obtained from the municipalities of Santa Leopoldina and Santa Maria de Jetibá in Espírito Santo, Brazil. The material comprised fresh, unprocessed rhizomes and rhizome fragments rejected during postharvest cleaning and grading because of mechanical damage, signs of disease or insect attack, or appearance unsuitable for export and domestic markets. Bananas (*Musa acuminata*) belonging to the Prata group (genomic group AAB) and free of pesticides were harvested from a farm in the municipality of Alegre, Espírito Santo, Brazil. The isolate *C. musae* (CCF 243), provided by the Department of Phytopathology of the Federal University of Viçosa, UFV Campus of Viçosa, was previously identified. Ginger rhizomes and bananas were transported to the Phytochemistry Laboratory at the Federal Institute, Campus Alegre, for subsequent processing and experimental setup.

### Extraction and determination of physicochemical properties of the FGEO

FGEO was extracted by hydrodistillation using a Clevenger-type apparatus. Four independent extractions were performed. In each extraction, 150 g of fresh ginger rhizome was ground in a blender for 2 min and transferred to a 2,000 mL distillation flask containing 1,000 mL of distilled water. After 4 h of hydrodistillation, each hydrolate was collected separately and centrifuged at 5,000 rpm for 10 min to obtain pure FGEO, which was stored in an amber flask under refrigeration. The extraction yield was calculated separately for each of the four independent extractions as the mass of FGEO obtained (g) divided by the mass of fresh rhizome used (150 g), multiplied by 100. The mean extraction yield of the four independent extractions was 0.21% (w/w). Photographic documentation of the extraction and analytical equipment is provided in Figures S1 and S2.

GC analysis of the FGEO was performed on a Shimadzu (GC 2012) Plus equipped with a flame ionization detector (GC-FID); for GC-FID analysis, a fused silica capillary column (30 m × 0.25 mm) with a DB5 stationary phase (0.25 μm film thickness) was used; the carrier gas was N2. For the calculation of retention indices (RI), a homologous mixture of alkanes (C7-C30) was used, and the values obtained for each compound were compared with those reported in the literature [16]. For GC–MS analyses, a Shimadzu (QPMS-2010) chromatograph was used, operating in electron-impact mode with an impact energy of 70 eV; scan speed of 1,000; scan interval of 0.50 fragments/second; detected fragments from 29 to 400 (m/z); helium (He) was used as the carrier gas. Compound identification was performed by comparing the compounds with the equipment libraries: NIST05, NIST05s, NIST12, NIST62, and Wiley 7.

### Evaluation of the antifungal activity of the FGEO by mycelial growth inhibition in vitro

Discs of *C. musae* were subcultured onto Potato Dextrose Agar (PDA) plates and incubated in a BOD incubator at 25 ± 1 °C under a 12 h light/dark photoperiod for 7 days. A freshly prepared stock emulsion was used in each independent experiment and contained 500 µL FGEO (5%, v/v), 100 µL Tween 80 (1%, v/v), and distilled water to a final volume of 10 mL. The emulsion was homogenized in an ultrasonic bath at 42 kHz for 45 min. Aliquots were diluted in PDA containing amoxicillin to obtain FGEO concentrations of 0.039, 0.078, 0.156, 0.313, 0.469, 0.625, 0.703, and 0.938 µL mL⁻¹. These concentrations were selected from preliminary tests to permit estimation of IC₅₀ and IC₉₀. The assay followed a completely randomized design and comprised three independent experiments, each with five Petri dishes per treatment. Thiabendazole (Tecto^®^) at 0.92 µL mL⁻¹ was used as the positive control. The negative control was prepared directly as a 1% (v/v) solution of Tween 80 in distilled water and did not undergo an additional dilution step. After solidification of the media, a 4 mm mycelial disc was placed at the center of each plate. The plates were sealed and incubated at 25 ± 1 °C under a 12 h light/dark photoperiod. Preparation of the stock emulsion and mycelial-growth assay are documented in Figures S3–S6.

### Calculation of the mycelial growth inhibition percentage of C. musae

To analyze the percentage mycelial growth inhibition for each concentration, diametrically opposite measurements of the colony halo were taken with a digital caliper calibrated in millimeters for each treatment replicate on the fifth (5th) day of incubation (the period at which the colony diameter reached the edge of the Petri dish in the negative control). The percentage of mycelial growth inhibition (PMGI) was determined using the equation described by Kordali et al. [17]:

$$PMGI\;\left(\%\right)\;=\;\frac{D_c-D_t}{D_c}\;\times\;100$$

where Dc = mean mycelial diameter of the negative control (mm) and Dt = mean diameter of the treatments (mm).

$$PMGI\;\left(\%\right)\;=\;\left[\left(Dc\;-\;Dt\right)/Dc\right]\;\times\;100$$

### Conidia germination inhibition test of C. musae

Mycelial discs of *C. musae* were subcultured on PDA and incubated at 25 ± 1 °C under a 12 h light/dark photoperiod. A conidial suspension was adjusted to 1 × 10⁶ conidia mL⁻¹, and 130 µL was spread on 3 cm Petri dishes containing water agar supplemented with FGEO at 0.039–0.938 µL mL⁻¹. The FGEO treatments were prepared from a freshly sonicated stock emulsion containing 5% (v/v) FGEO and 1% (v/v) Tween 80. Thiabendazole (0.92 µL mL ⁻¹) was used as the positive control, whereas the negative control was prepared directly as 1% (v/v) Tween 80 in distilled water without an additional dilution step. The treatments were incubated in the dark at 25 ± 1 °C for 9 h. Each plate was examined under a Leica DM 500 biological microscope, and 100 conidia were evaluated per replicate for each treatment. Conidial germination inhibition was calculated as (inhibited conidia/total conidia) × 100. Additional representative micrographs and the complete inhibition data are provided in Figure S7 and Table S1, respectively.

### Effect of the FGEO on C. musae

The fungicidal or fungistatic effect of FGEO on *C. musae* was analyzed by transferring discs containing fungal mycelia that had been in contact with the most effective oil concentrations in the mycelial growth inhibition test. Thus, 4 mm discs were removed from these colonies and transferred to new PDA culture media. The same was done for treatments containing positive and negative controls. Subsequently, after 5 days, the presence or absence of mycelial growth was analyzed. Each treatment had five replicates.

### Evaluation in vivo

Banana bunches were harvested at ripeness stage 2 of the Von Loesecke scale [18]. To ensure maximum uniformity, fruits from the center of the bunch were used, with the Dole^®^ color guide (Dole Food Company, Inc., CA, USA) [19] as the selection parameter. The fruits were stored in the laboratory at 16 °C for 2 days (the period at which they reached ripeness stage 3 according to the Von Loesecke scale [18]). After reaching ripeness stage 3, bananas were washed with neutral detergent in running water; their surfaces were then disinfected with 2% sodium hypochlorite for 5 min, rinsed with distilled water, and placed on two paper towel sheets to dry at 23 °C for 3 h.

Five FGEO concentrations were evaluated in vivo: 0.201, 0.401, 0.803, 1.606, and 3.212 µL mL⁻¹. These concentrations were selected around the IC₉₀ estimated in the in vitro mycelial growth assay, with two concentrations below and two above this value. This range was adopted because oil bioavailability in the fruit matrix, volatilization, and host physiological responses may modify antifungal performance under in vivo conditions. A 1% (v/v) Tween 80 solution in distilled water, prepared directly without dilution, was used as the negative control, and thiabendazole at 0.92 µL mL⁻¹ was used as the positive control. Each treatment comprised four replicates, with two banana fingers per replicate.

Three wounds were created on each banana finger. Each wound received 10 µL of a 7-day-old *C. musae* conidial suspension adjusted to 1 × 10⁶ conidia mL⁻¹. After 24 h, 10 µL of the corresponding FGEO, negative control, or positive control was applied to each wound. The FGEO stock emulsion was diluted in distilled water to obtain the evaluated concentrations, whereas the negative control solution was prepared directly and not diluted. The fruits were incubated at 25 ± 1 °C under a 12 h light/dark photoperiod. Lesion diameter was measured after 5 days, and inhibition was calculated using the equation applied in the mycelial growth assay. The in vivo assay comprised two independent experiments conducted under a completely randomized design. The in vivo experimental setup is illustrated in Figure S8.

### Statistical analyses

The homogeneity of variances was assessed using Levene’s test, and the normality of residuals was evaluated using the Shapiro–Wilk test. When both assumptions were met (*P* > 0.05), the data were subjected to analysis of variance. Simple linear regression was fitted to the in vitro concentration–response data, whereas logarithmic regression was fitted to the in vivo data to estimate IC₅₀ and IC₉₀. Model selection was based on the highest coefficient of determination (R²) and significance of the equation parameters (*P* < 0.05). FGEO concentrations were compared with those of the positive control using Dunnett’s test in the in vitro assays, whereas means from the in vivo assay were compared using Tukey’s test. All analyses were performed using R version 4.3.1.

## Results

### Physicochemical analysis of the fresh ginger essential oil

**Fig. 1:**
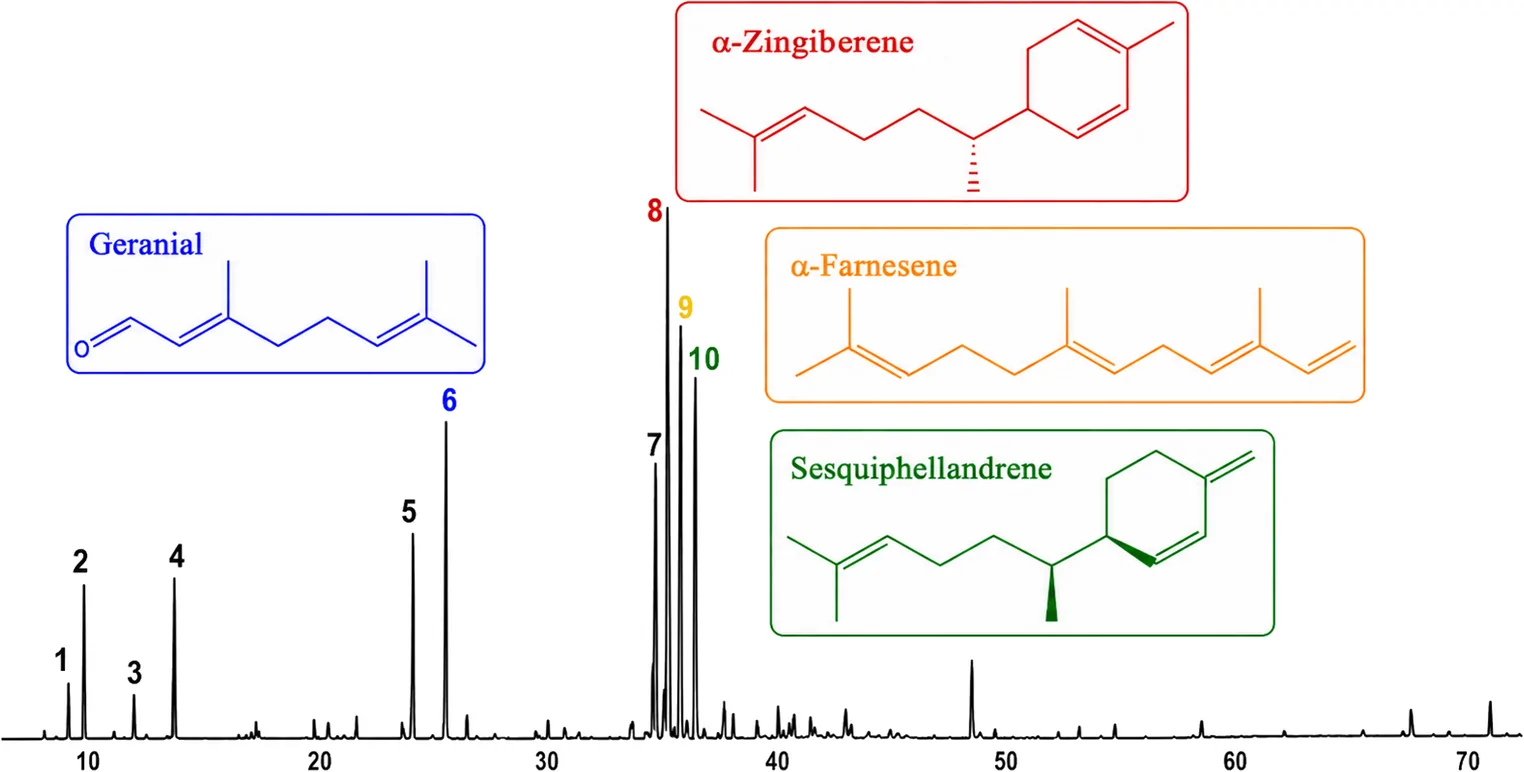
Chromatogram (GC-MS) of the ginger essential oil (*Z. officinale*) with retention time and chemical structure of the major compounds

Chemical characterization of FGEO revealed 10 identified compounds and an unidentified fraction representing 1.39% of the total chromatographic area. α-Zingiberene was the predominant compound (31.73%), followed by α-farnesene (14.85%), geranial (13.15%), and sesquiphellandrene (11.04%). The other identified compounds accounted for 27.84% of the relative area. Hydrocarbon sesquiterpenes were the predominant chemical class, accounting for 66.32% of the total relative area (Table 1). Figure 1 shows the chromatogram and chemical structures of the predominant compounds. The mean extraction yield was 0.21% (w/w).

**Table 1:** Characterization of ginger essential oil by the LTPRI index and GC-MS ^[a]^

Peak	RT^[b]^	IR^[c]^	IR^[d]^	Compound	Area (%)^[e]^
1	8.835	926	932	α-pinene	1.35
2	9.437	939	946	Camphene	4.11
3	11.495	984	988	β-myrcene	1.05
4	13.152	1020	1025	β-phellandrene	6.39
5	23.640	1227	1235	Neral	6.25
6	25.104	1257	1264	Geranial	13.15
7	34.336	1471	1479	α-Curcumene	8.70
8	34.917	1488	1493	α-Zingiberene	31.73
9	35.424	1500	1505	α-Farnesene	14.85
10	36.055	1515	1521	Sesquiphellandrene	11.04
	Unidentified				1.39
	Hydrogenated monoterpenes				12.90
	Oxygenated monoterpene				19.40
	Hydrogenated sesquiterpene				66.32
	Oxygenated sesquiterpene				-

[a] Compounds identified by the LTPRI index and GC-MS using an Rtx®-5MS column

[b] Retention time

[c] Calculated using a mixture of saturated n-alkanes (C7 to C40)

[d] Tabulated indices based on [16]

[e] Only compounds with a relative area > 1%

### In vitro inhibition of *C. musae* mycelial growth

**Fig. 2:**
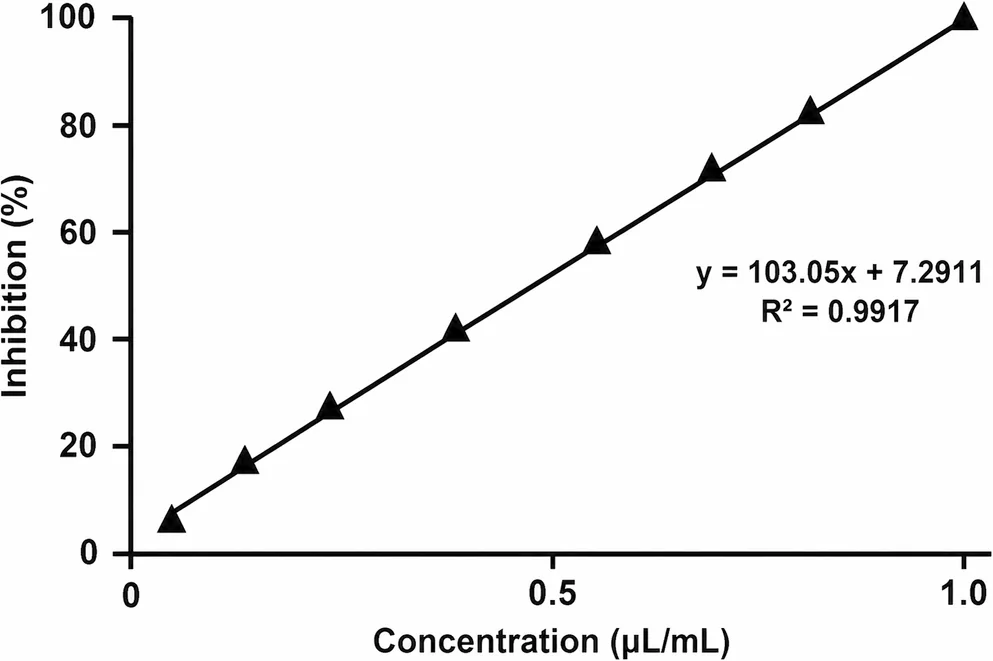
Percentage of mycelial growth inhibition of *C. musae* as a function of different FGEO treatments

The essential oil demonstrated a significant inhibitory effect (*P* < 0.05) on the mycelial growth of the fungus *C. musae*. The inhibitory concentrations IC₅₀ and IC₉₀ were estimated at 0.414 µL mL⁻¹ and 0.803 µL mL⁻¹, respectively (Fig. 2). The percentage inhibition of mycelial growth increased with increasing dose. A dose-dependent relationship was observed (Fig. 2; Figure S9; Table S2).

**Fig. 3:**
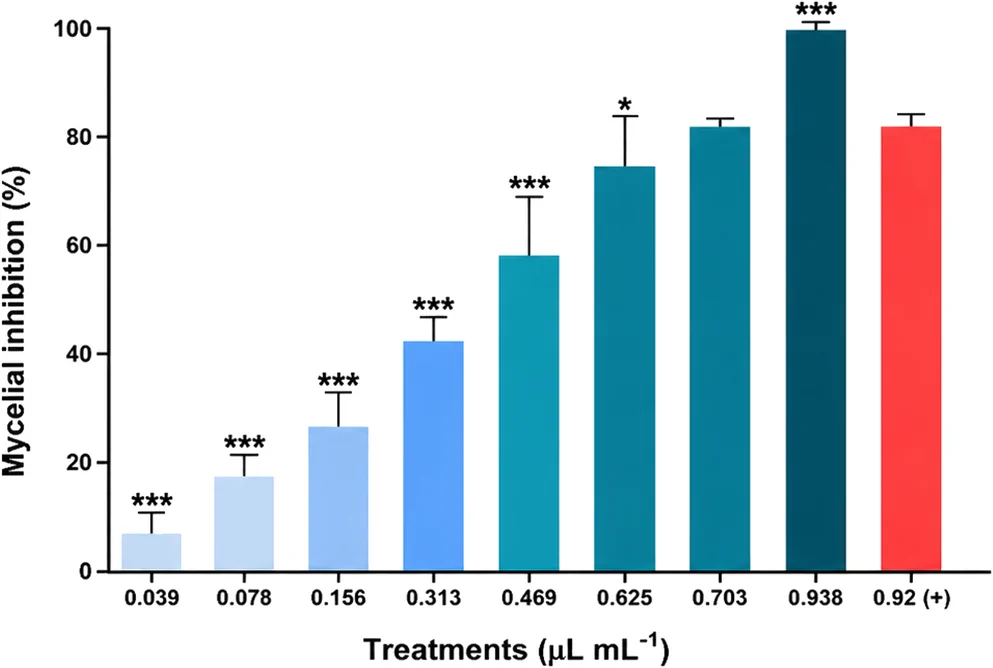
Effects of different FGEO concentrations and the positive control (thiabendazole) on the mycelial growth of *C. musae*. The bars represent the mean ± standard deviation (SD). Asterisks indicate significant differences from the positive control according to Dunnett’s test (*** *P* < 0.001, ** *P* < 0.01, and * *P* < 0.05)

FGEO at 0.938 µL mL⁻¹ resulted in approximately 100% inhibition of *C. musae* mycelial growth. The positive control, thiabendazole, applied at the manufacturer’s recommended concentration, resulted in 81.70% inhibition. FGEO at 0.703 µL mL⁻¹ resulted in 81.87% inhibition and did not differ significantly from the positive control, according to Dunnett’s test (Fig. 3).

### Conidia germination inhibition test of C. musae

**Fig. 4:**
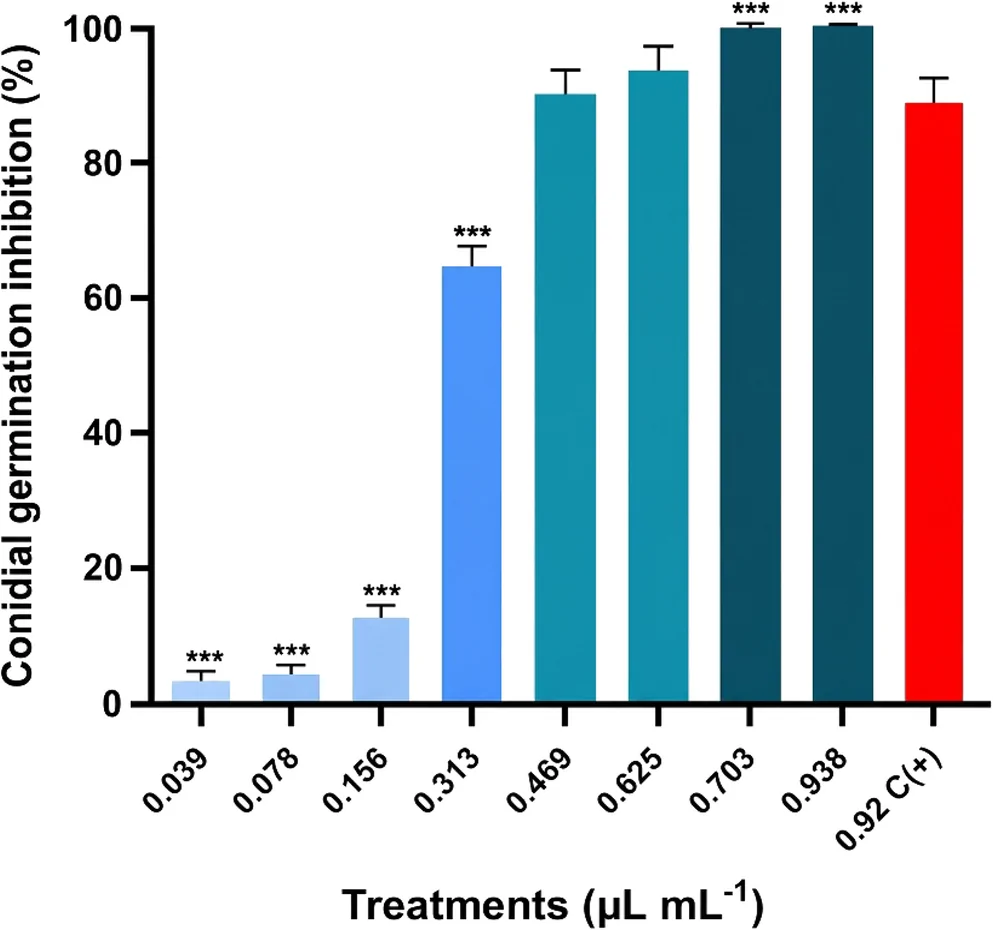
Effects of different FGEO concentrations and the positive control (thiabendazole) on the germination of *C. musae* conidia. The bars represent the mean ± standard deviation (SD). Asterisks indicate significant differences from the positive control according to Dunnett’s test (*** *P* < 0.001, ** *P* < 0.01, and * *P* < 0.05)

**Fig. 5:**
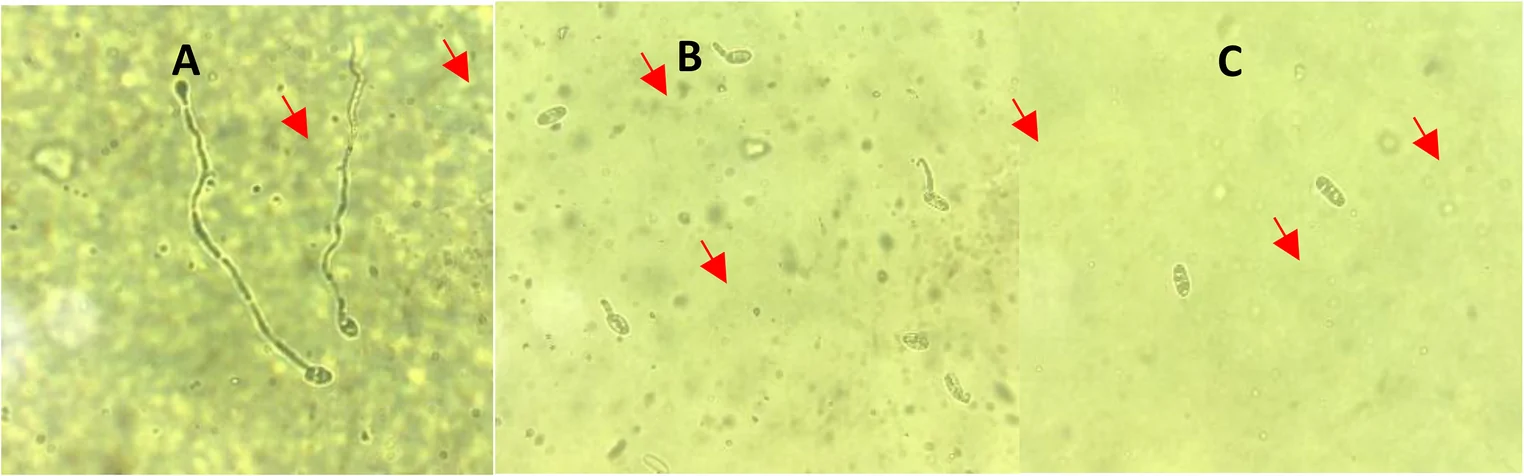
Effect of different treatments on conidia germination of *C. musae*. Light microscopy of *C. musae* conidia at 40x magnification. **A** – Negative control (distilled water + 1% Tween 80); **B** – Positive control (Thiabendazole); **C** – 0.938 µL mL^− 1^ FGEO

FGEO inhibited the germination of *C. musae* conidia in a concentration-dependent manner. The effects observed at 0.469 and 0.625 µL mL⁻¹ did not differ significantly from that of the positive control. FGEO at 0.703 and 0.938 µL mL⁻¹ completely inhibited conidial germination and showed significantly greater inhibition than that of the positive control (Figs. 4 and 5).

### Effect of the FGEO on anthracnose development in vivo

**Fig. 6:**
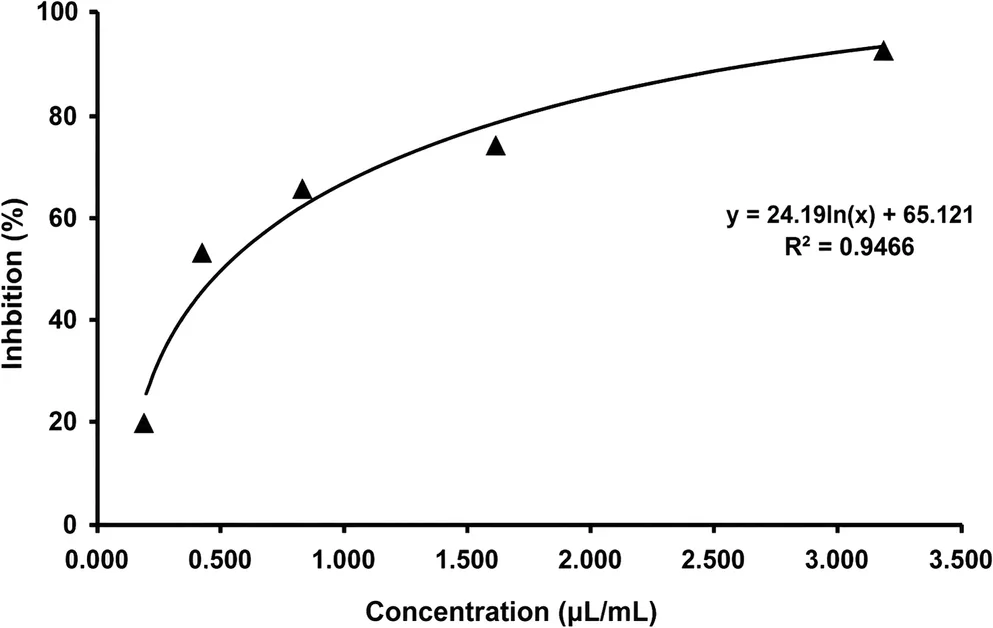
Percentage inhibition of anthracnose development in banana as a function of different FGEO treatments under in vivo conditions

**Fig. 7:**
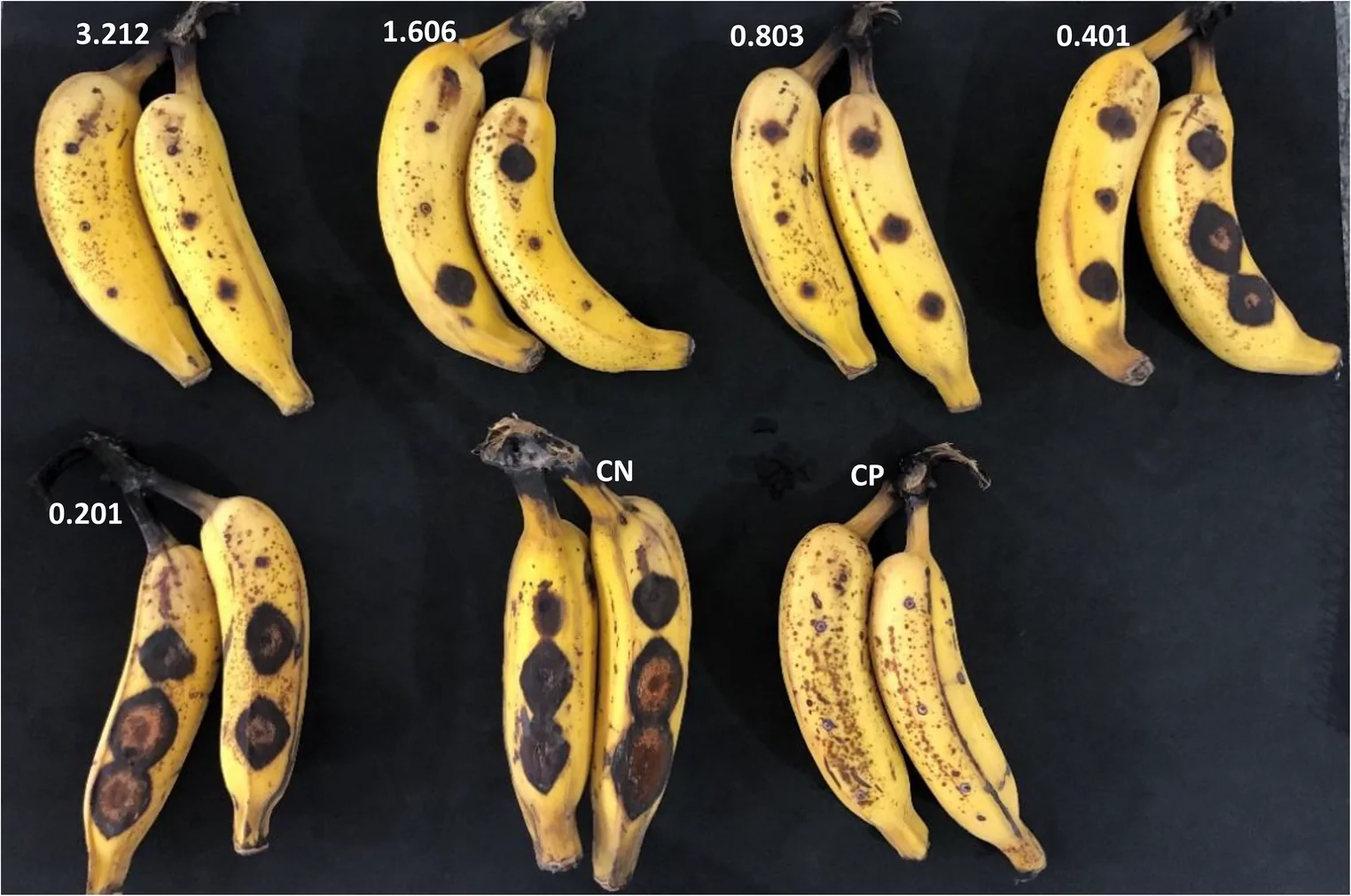
Effect of different FGEO treatments (µL mL-1), negative control (CN), and positive control (CP thiabendazole) on the inhibition of banana anthracnose

FGEO inhibited the development of banana anthracnose under in vivo conditions (*P* < 0.05) (Figs. 6 and 7), with an IC_50_ of 0.535 µL mL-1 and an IC_90_ of 2.797 µL mL^− 1^.

All bananas treated with different FGEO concentrations differed from the negative control according to Tukey’s test. The concentration of 3.212 µL mL-1 inhibited 92.29% of anthracnose development up to the fifth day of inoculation, not differing statistically from the positive control, which under in vivo conditions achieved 100% inhibition. The intermediate concentrations also showed significant inhibition percentages (Table 2). Representative fruits are shown in Figure S10.

**Table 2:** Inhibition percentage of different FGEO concentrations, negative control, and positive control on banana anthracnose development

Concentration (µL mL^− 1^)	% Inhibition
3.212	92.29^a^ ± 5.14
1.606	73.11^b^ ± 2.68
0.803	62.60^b^ ± 10.48
0.401	52.78^b^ ± 16.76
0.201	19.78^c^ ± 6.97
C-	0^d^ ± 0
C+	100^a^ ± 0

Means followed by the same letter in the column do not differ statistically using Tukey’s test at 5% probability. C- (negative control): 1% (v/v) Tween 80 in distilled water; C+ (positive control): Tecto Fungicide at a dose of 0.92 µL mL^− 1^

## Discussion

The present study demonstrated that fresh ginger production waste can provide an essential oil with antifungal activity against *C. musae*, linking the valorization of post-harvest residues with the potential control of banana anthracnose. Essential oils are complex mixtures with diverse applications. They consist of terpenic compounds, such as monoterpenes (compounds formed by the union of two isoprene units) and sesquiterpenes (compounds formed by the union of three isoprene units) [20].Chemical analysis of fresh ginger essential oil obtained from ginger production waste showed that the major compound was α-zingiberene (31.73%). This compound was evaluated for its cytotoxic activity against tumor cell lines and showed positive results [21]. In Lee [22], α-zingiberene induced apoptosis in SiHa cells and showed promising potential for treating chronic diseases [23].

The other predominant compounds identified in the FGEO were α-farnesene (14.85%), geranial (13.15%), and sesquiphellandrene (11.04%). Compounds present individually at lower relative abundances collectively accounted for 27.84% of the oil and should not be regarded as biologically negligible because constituents of complex essential oils may contribute through additive or synergistic interactions. Previous studies have reported both similarities and substantial variations in ginger essential oil composition [24]. The predominance of α-zingiberene (31.73%) is relevant to the chemical characterization of FGEO; however, the present assays evaluated the whole oil rather than isolated constituents. Consequently, the antifungal response cannot be attributed exclusively to α-zingiberene, geranial, α-farnesene, or sesquiphellandrene. Identified and unidentified constituents may have contributed through independent, additive, or synergistic effects. Essential oil constituents have been reported to affect fungal membranes, ergosterol-related processes, and cellular energy metabolism [25, 26]; however, these mechanisms were not investigated here and should be regarded as hypotheses for FGEO.

Poudel et al. [27] also worked with FGEO from samples collected at 12 different locations and identified compounds similar to those characterized in this study, but in different proportions. For example, α-zingiberene ranged from 0.6% to 11.8%, geranial from 1.0% to 17.4%, and sesquiphellandrene from 3.7% to 9.7%. In the work by Ibáñez and Blázquez [28], α-zingiberene accounted for 24.9% of the relative area and was the main compound identified; the other major compounds were β-sesquiphellandrene (11.9%), ar-curcumene (10.7%), and β-bisabolene (10.5%). The yield of ginger essential oil can also vary [29]. In the work by Prasath et al. [30], using dried rhizomes, the yield was 1.20% w/w. The characteristics of ginger essential oil are sensitive to various factors. Edaphoclimatic factors, as well as storage conditions, rhizome pre-treatment, and extraction methods, directly influence the yield, color, and chemical composition of the essential oil [29, 31, 32]. An important aspect of the present study is that FGEO was obtained from fresh ginger production waste generated during postharvest cleaning and grading. Despite being rejected for commercial purposes, this material yielded an essential oil rich in α-zingiberene, α-farnesene, geranial, and sesquiphellandrene, and with relevant antifungal activity. These findings demonstrate the potential of ginger production waste as a source of bioactive essential oil, providing an alternative for the valorization of material that would otherwise be discarded or left as organic residue in production areas.

In addition, this study demonstrated the antifungal activity of FGEO against *C. musae* under both in vitro and in vivo conditions. FGEO inhibited mycelial growth and conidial germination in a concentration-dependent manner and reduced disease development in banana fruits. For mycelial growth, the estimated IC₅₀ and IC₉₀ values were 0.414 and 0.803 µL mL⁻¹, respectively, whereas in vivo the corresponding values were 0.535 and 2.797 µL mL⁻¹, respectively. The antifungal activity of ginger essential oil has also been reported against other phytopathogenic fungi. Abdullahi et al. [14] evaluated a ginger essential oil containing α-zingiberene (18.56%) and geranial (13.88%) against *Fusarium oxysporum*, *Pyricularia oryzae*, *Colletotrichum falcatum*, *Ganoderma boninense*, and *Rigidoporus microporus*, reporting complete inhibition at concentrations ranging from 5 to 6 µL mL⁻¹. Other essential oils have also shown activity against *C. musae.* Essential oils from *Ocimum basilicum* (estragole, 7.4%) and *Melaleuca alternifolia* (eucalyptol, 57.02%) inhibited *C. musae* growth in vitro at 5 and 4.5 µL mL⁻¹, respectively, and delayed anthracnose severity in vivo [33].

Batista et al. [34] evaluated the inhibitory potential of the leaf essential oil of *P. macedoi* (piperone 26.48%, sylvestrene 13.03%, and bicyclogermacrene 10.45%) on the mycelial growth of *C. musae*. According to the authors, at low concentrations, the essential oil exhibited an antifungal effect, with IC_90_ and IC_50_ values of 1.80 µL mL^− 1^ and 0.804 µL mL^− 1,^ respectively. Gonçalves et al. [35] evaluated the antifungal activity of M. alternifolia essential oil (terpinen-4-ol 47.08%, γ-terpinene 22.33%, and α-terpinene 10.90%) against *C. musae*. The authors observed a significant increase in fungal halo inhibition as the essential-oil concentration increased. The IC_50_ was estimated at 0.42 µg mL^− 1^ and IC_100_ were estimated to be 0.42 and 2.15 µg mL − 1, respectively. In vivo, the incidence and severity of banana diseases were reduced.

The essential oil of *M. fistulosa* (thymol 23.43%, carvacrol 23.43%, and cinnamyl carbonate 17.54%) inhibited 100% of the mycelial growth of *C. musae* and reduced the severity of anthracnose in vivo. The authors attributed the antifungal effect to the major compounds present in the essential oil [36]. The botanical species *Cinnamomum cassia* (trans-cinnamaldehyde 39.39%, caryophyllene 18.95%, and eugenol 12.44%) and *Ocimum tenuiflorum* (eugenol 27.56%, caryophyllene 27.56%) showed antifungal effects against *C. musae*; the authors reported that the probable compounds responsible for the antifungal action include major ones such as eugenol and cinnamyl acetate, as well as some minor compounds such as humulene and trans-calamenene, which may also influence biological activity [37].

The complexity of essential oils allows them to interfere with cellular metabolism in different ways. Researchers have been investigating the target sites and mechanisms of action of essential oils that inhibit or cause cell lysis, since, unlike traditional fungicides, this information is not yet fully elucidated, although some data are already available [25]. There are reports that essential oils interfere with ergosterol synthesis [26] and inhibit the synthesis of the enzyme chitin synthase [36]. Furthermore, due to their lipophilic nature, essential oils can cross the cell membrane of the phytopathogen and reach the mitochondria, thereby impairing the energy production of the cell [25]. In the in vivo test, a higher ginger essential oil concentration was needed to inhibit the growth of *C. musae* compared to the in vitro. Factors that may explain the need for higher essential oil concentrations in vivo include the fact that, on the banana surface, the oil must penetrate cell layers and interact with natural fruit compounds, such as waxes and metabolites, which may interfere with its bioavailability [38].

However, these concentration adjustments do not invalidate the efficacy of the product, as the FGEO application in vivo significantly reduced anthracnose development in banana, achieving 92.29% inhibition at a concentration of 3.212 µL mL⁻¹, a result statistically similar to that of the commercial fungicide. It is also noteworthy that the FGEO concentration used under in vivo conditions test in this study remains lower than that used in other studies employing essential oils for anthracnose control [33]. Based on the reported findings, the use of essential oils to control postharvest diseases such as anthracnose is an emerging and promising alternative [39]. Continuing research in this field is essential to integrate an approach to the phytosanitary management of agricultural crops that promotes the reduction of synthetic chemical products, ensures food safety, and environmental sustainability [4, 5].

## Conclusion

Fresh ginger (*Zingiber officinale*) essential oil, obtained from ginger production waste, inhibited the mycelial growth and conidial germination of *Colletotrichum musae* and reduced anthracnose development in banana fruits under the evaluated conditions. By integrating chemical characterization with in vitro and in vivo assays, this study provides evidence to support further investigation of ginger production waste as a source of antifungal products for postharvest management. As the entire oil was tested, its activity should be interpreted as the combined effect of its constituents rather than the effect of a single compound. Further studies should assess formulation stability, safety, residues, performance under commercial storage conditions, extraction scale-up, and economic feasibility.

## Supplementary Information

Below is the link to the electronic supplementary material.

Supplementary Material 1 (DOCX 11.3 MB)
