# Supplementary material for: Antifungal activity of fresh ginger (Zingiber officinale) essential oil from ginger production waste against banana anthracnose caused by Colletotrichum musae: in vitro and in vivo evaluation
Source: Braz J Microbiol. 2026 Oct 8;57(1):286. doi: 10.1007/s42770-026-02108-2 (PMC13650211; doi:10.1007/s42770-026-02108-2)
Supplement: Supplementary file 1 [file 42770_2026_2108_MOESM1_ESM.docx]

**Supplementary Material**

**Antifungal activity of fresh ginger (*Zingiber officinale*) essential oil from ginger production waste against banana anthracnose caused by *Colletotrichum musae*: *in vitro* and *in vivo* evaluation**

Maria Alice Brandão Silva^1^, Aldino Neto Venâncio^1^, Isabella da Costa Teixeira^1^, Ana Paula Candido Gabriel Berilli^1^, Sávio da Silva Berilli^1^, Luciana Alves Parreira^2^, Mario Ferreira Conceição Santos*^2^, Antônio Fernando de Souza^3^, Luciano Menini*^1^

*^1^Graduate Program in Agroecology, Federal Institute of Espírito Santo, Campus Alegre, Alegre – Espírito Santo, Brazil*

*^2^ Federal Institute of Espírito Santo, Campus Santa Tereza, Santa Tereza – Espírito Santo, Brazil*

*^3^Department of Chemistry and Physics, Federal University of Espírito Santo, Alegre – Espírito Santo, Brazil*

***Corresponding authors:** Mario Ferreira Conceição Santos ([mariosantos408@gmail.com](mailto:mariosantos408@gmail.com)); Luciano Menini (lmenini@ifes.edu.br)

**D**

**A**

**B**

**C**

**Figure S1.** A: Fresh ginger, cut and weighed; B: Fresh ginger being ground in a blender; C: FGEO extraction in a Clevenger apparatus; D: FGEO in storage flask.

**A**

**B**

**Figure S2.** A: Freezer where FGEO was stored; B: GC-FID: One of the instruments used for chemical analysis of the FGEO.

**B**

**A**

**Figure S3.** A: Components used to prepare the FGEO stock emulsion: 500 µL FGEO, 100 µL Tween 80, and distilled water to a final volume of 10 mL; B: Stock emulsion being homogenized in an ultrasonic bath at 42 kHz for 45 min.

**B**

**A**

**B**

**Figure S4.** A: Emulsion analysis; B: Ready emulsion.

**B**

**A**

- **B**

**Figure S5.** A: FGEO stock emulsion being collected for dilution to the experimental concentrations; B: Culture medium containing the FGEO treatment being poured into a Petri dish. The negative control was prepared directly as 1% (v/v) Tween 80 in distilled water and did not undergo an additional dilution step.

**A**

**B**

**C**

**Figure S6.** A: Mycelial discs of *C. musae* being cut; B: Discs of *C. musae* being inoculated at the center of the Petri dish; C: Petri dishes with treatments and *C. musae* being stored in BOD incubator.

**A**

**B**

**C**

**D**

**E**

**F**

**G**

**H**

**I**

**J**

**Figure S7.** Effect of different FGEO concentrations, negative control (C-), and positive control (C+) on conidia germination inhibition of *C. musae* after 9 hours in the BOD incubator, observed under a Leica DM 500 biological microscope at 40× magnification. A: 0.039; B: 0.078; C: 0.156; D: 0.313; E: 0.469; F: 0.625; G: 0.703; H: 0.938 µL mL⁻¹; I: (C-); J: (C+).

**B**

**C**

**A**

**Figure S8**. A: Banana finger at ripeness stage 2 of the Von Loesecke scale; B: Banana at ripeness stage 3, sanitized and inoculated with 10 µL of a *C. musae* suspension adjusted to 10⁶ conidia mL⁻¹; C: Trays containing inoculated bananas treated with FGEO, the negative control (1% Tween 80 in distilled water), or the positive control and incubated in a BOD chamber.

0.703

0.938

0.625

0.469

0.313

0.156

0.078

0.039

C-

C+

**Figure S9.** Effect of different FGEO treatments, negative control (C-), and positive control (C+ thiabendazole) on the inhibition of mycelial growth of *C. musae* in µL mL^⁻¹^ after 5 days of incubation.

**A**

**B**

**Figure S10.** Banana inoculated with *C. musae* conidia after 5 days. A: Fruit treated with 3.212 µL mL⁻¹ FGEO; B: Negative control, consisting of 1% (v/v) Tween 80 in distilled water.

**Table S1.** Effect of FGEO and positive control (Thiabendazole) on the conidia germination inhibition percentage of *C. musae.*

| Concentration (µL mL⁻¹) | % Inhibition |
| --- | --- |
| 0.938 | 100.00 |
| 0.703 | 99.2 |
| 0.625 | 92.2 |
| 0.469 | 88.6 |
| 0.313 | 65.4 |
| 0.156 | 12.4 |
| 0.078 | 3.0 |
| 0.039 | 2.2 |
| C (+) | 89.6 |

Means were compared with the positive control using Dunnett’s test (*** P < 0.001, ** P < 0.01, * P < 0.05), (CV: 5.06%)

**Table S2.** Effect of FGEO and positive control (Thiabendazole) on the mycelial growth inhibition percentage of *C. musae.*

| Concentration (µL mL⁻¹) | % Inhibition |
| --- | --- |
| 0.938 | 100.00 |
| 0.703 | 81.87 |
| 0.625 | 72.65 |
| 0.469 | 57.44 |
| 0.313 | 41.69 |
| 0.156 | 24.71 |
| 0.078 | 16.64 |
| 0.039 | 5.56 |
| C (+) | 81.70 |

Means were compared with the positive control using Dunnett’s test (*** P < 0.001, ** P < 0.01, * P < 0.05), (CV: 13.08%).
